# Antibody targeting of the TNF–TNFR2 axis to overcome tumor immune resistance

**DOI:** 10.3389/fimmu.2026.1809305

**Published:** 2026-05-14

**Authors:** Xiaozhen Kang, Xiangmin Tong, Shibing Wang

**Affiliations:** 1 Zhejiang Key Laboratory of Zero Magnetic Medicine, Affiliated Hangzhou First People’s Hospital, School of Medicine, Westlake University, Hangzhou, Zhejiang, China; 2 Department of Clinical Laboratory, Affiliated Hangzhou First People’s Hospital, School of Medicine, Westlake University, Hangzhou, Zhejiang, China

**Keywords:** antibody, immune resistance, immunotherapy, TNF, TNFR2

## Abstract

Tumor necrosis factor (TNF) exerts paradoxical effects in cancer, driven by the differential engagement of its two receptors, TNFR1 and TNFR2. While TNFR1 mediates cytotoxic signaling, accumulating evidence indicates that TNFR2 predominantly orchestrates tumor-promoting inflammation and immunosuppression within the tumor microenvironment (TME). TNFR2 is highly expressed on regulatory T cells (Tregs), myeloid-derived suppressor cells (MDSCs), cancer-associated fibroblasts (CAFs), and malignant cells, forming a coordinated network that drives immune evasion and contributes to resistance to immune checkpoint blockade (ICB). In this review, we dissect the structural and functional distinctions between transmembrane and soluble TNF and discuss how these differences shape receptor-specific signaling outcomes. We further highlight emerging therapeutic strategies targeting the TNF–TNFR2 axis, including monoclonal antibodies, antibody–drug conjugates (ADCs), and bispecific antibodies, with an emphasis on their ability to selectively remodel the immunosuppressive TME. Finally, we discuss key challenges for clinical translation, including on-target toxicity, patient stratification, and context-dependent TNFR2 biology, and outline future directions such as biomarker-guided therapy and tumor-restricted targeting approaches. Together, these advances position TNFR2 as a promising therapeutic node for overcoming resistance to current immunotherapies.

## 1 Introduction

TNF is a central inflammatory cytokine that plays multifaceted and context-dependent roles in cancer. Originally identified for its tumoricidal activity, TNF is now recognized as a critical regulator of tumor-promoting inflammation, immune suppression, and therapeutic resistance (1, 2). In the TME, sustained TNF signaling contributes to cancer progression by shaping immune cell composition, promoting angiogenesis, and fostering chronic inflammation (3, 4).

Importantly, accumulating evidence suggests that TNF signaling is intimately involved in limiting the efficacy of modern cancer immunotherapies, particularly antibody-based immune checkpoint blockade (5, 6). Studies have revealed that TNF can paradoxically undermine antitumor immune responses by reinforcing immune tolerance (1). For instance, macrophage-derived TNF has been shown to induce resistance to anti-PD-1 therapy by promoting the apoptosis of CD8^+^ T cells and stabilizing PD-L1 expression on tumor cells, highlighting the need for therapeutic strategies that can selectively modulate TNF signaling without broadly suppressing antitumor immunity (5, 7, 8).

The biological complexity of TNF signaling is largely determined by its engagement of two structurally and functionally distinct receptors, TNFR1 and TNFR2. While TNFR1 is ubiquitously expressed and contains an intracellular death domain (DD) that mediates pro-apoptotic and necroptotic signaling pathways, TNFR2 lacks a DD (9). Instead, TNFR2 exhibits a more restricted expression pattern and predominantly activates cell survival and proliferation pathways via NF-κB and PI3K-Akt (10). This receptor-specific signaling dichotomy provides a mechanistic framework for understanding how TNF can simultaneously exert cytotoxic antitumor effects (via TNFR1) and promote tumor survival and immune suppression (via TNFR2). Given the increasing clinical reliance on antibody-based cancer immunotherapies, there is growing interest in exploiting this receptor-selective approach. Rather than globally neutralizing TNF, which may lead to systemic toxicity and dampen baseline antitumor immunity, targeting the TNF–TNFR2 axis offers the potential to selectively dismantle immunosuppressive networks within the TME (11).

While several excellent reviews published in recent years have comprehensively summarized the fundamental biology of TNFR2 and its canonical role in Treg-mediated immunosuppression, this review is specifically designed to bridge the gap between receptor kinetics, emerging TME networks, and therapeutic engineering. Here, we distinctly focus on how the structural kinetics of tmTNF versus sTNF dictate rational drug design. Furthermore, we expand the conventional scope to highlight the critical, self-amplifying roles of CAFs and tumor-intrinsic TNFR2 signaling. Most importantly, we provide a critical evaluation of next-generation, multi-modal targeting strategies—specifically detailing the structural and translational considerations of bispecific antibodies, ADCs, and localized delivery vehicles like engineered oncolytic adenoviruses. By synthesizing these advances, this review offers a definitive roadmap for overcoming the limitations of early-stage monoclonal therapies and advancing TNFR2-targeted interventions toward clinical reality.

## 2 Genetics, structure, and kinetics of TNF/TNFR interactions

**Figure 1:**
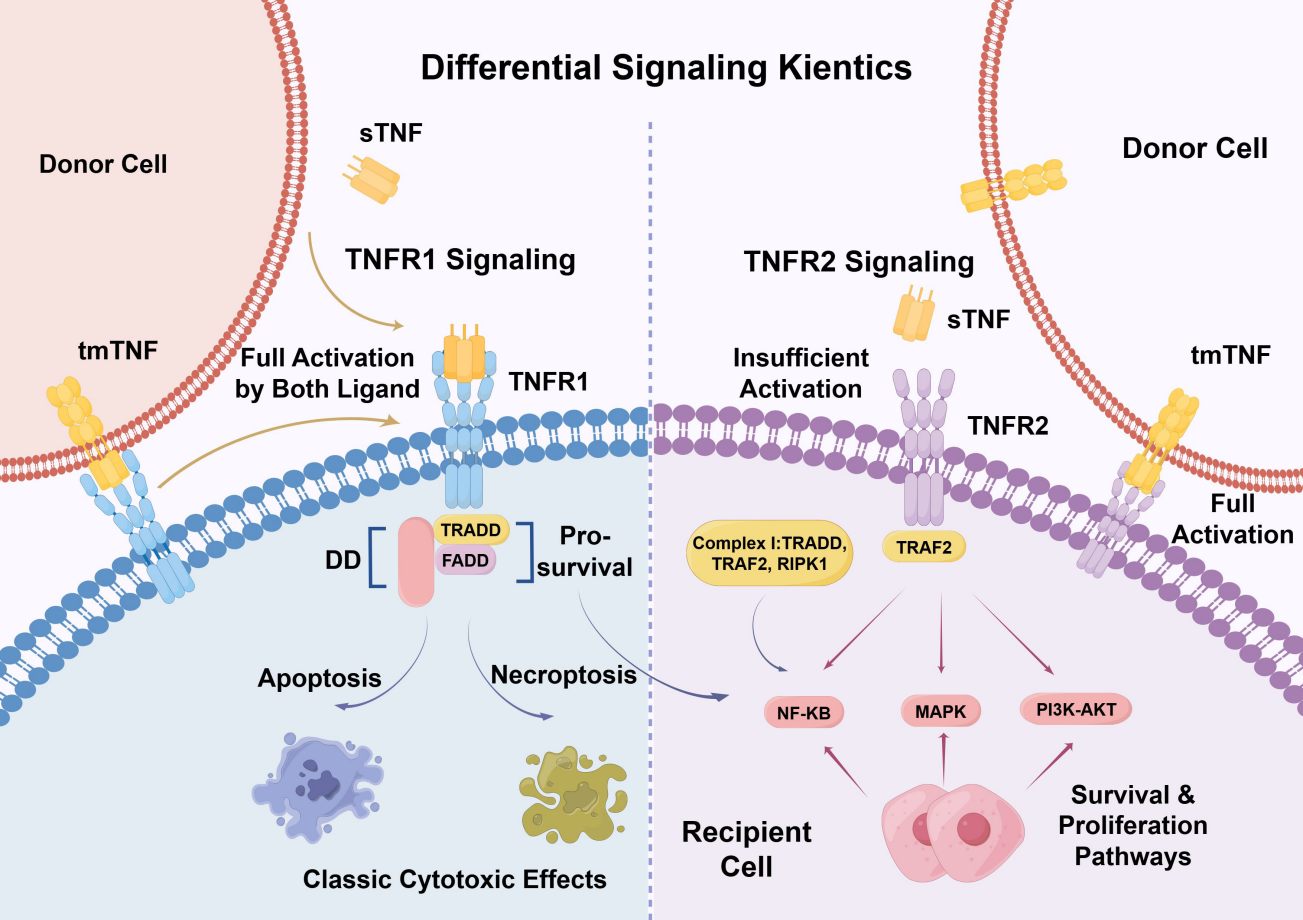
Differential signaling kinetics of the TNF/TNFR axis. Differential signaling kinetics and cellular outcomes. TNFR1 can be fully activated by both sTNF and tmTNF. Its intracellular DD recruits TRADD and FADD, capable of initiating pro-survival signals via Complex I (NF-κB) or driving classic cytotoxic effects through caspase-8-mediated apoptosis and RIPK1-mediated necroptosis. Conversely, optimal activation of TNFR2 strictly requires direct cell-to-cell contact mediated by tmTNF, which drives robust receptor clustering. While sTNF can bind TNFR2, it is generally insufficient for full activation. Lacking a DD, TNFR2 directly recruits TRAF2 to potently initiate survival and proliferation cascades, including the NF-κB, MAPK, and PI3K-AKT pathways.

To effectively target the TNF signaling axis, it is crucial to understand the genetic and molecular distinctions between TNF ligands and their receptors. The gene encoding TNF is located within the major histocompatibility complex (MHC) class III region on chromosome 6 (12). TNF is initially synthesized as a 26 kDa transmembrane precursor protein (tmTNF) (13). This membrane-bound form can be proteolytically cleaved by TNF-alpha converting enzyme (TACE/ADAM17) to release a 17 kDa soluble TNF (sTNF) molecule (14). Both tmTNF and sTNF assemble into non-covalent homotrimers to exert their biological functions, but they interact with TNFR1 and TNFR2 differently (**Figure 1**) (15).

TNFR1 is ubiquitously expressed across nearly all cell types and can be fully activated by both sTNF and tmTNF. Upon binding, the intracellular DD of TNFR1 recruits adapter proteins such as TRADD and FADD, which can subsequently trigger caspase-8-mediated apoptosis or RIPK1/3-mediated necroptosis, underlying the classic cytotoxic effects of TNF (16–18).

In contrast, TNFR2 expression is highly regulated and primarily restricted to immune cells, endothelial cells, and certain tumor cells (19). Crucially, while sTNF can bind to TNFR2, it is generally insufficient to fully activate the receptor. Optimal activation of TNFR2 requires the binding of tmTNF, which facilitates robust receptor clustering and the recruitment of TRAF2 (TNF receptor-associated factor 2), initiating potent NF-κB, MAPK, and PI3K–AKT signaling cascades (20). This dependence on tmTNF restricts TNFR2 signaling to cell–cell contact contexts. Consequently, the design of anti-TNFR2 antibodies must carefully account for whether the therapeutic goal is to block tmTNF binding, lock the receptor in an inactive conformation, or deplete TNFR2-expressing cells entirely.

## 3 TNFR2 as a key regulator of the Immunosuppressive TME: Tregs, MDSCs, CAFs, and tumor cells

**Figure 2:**
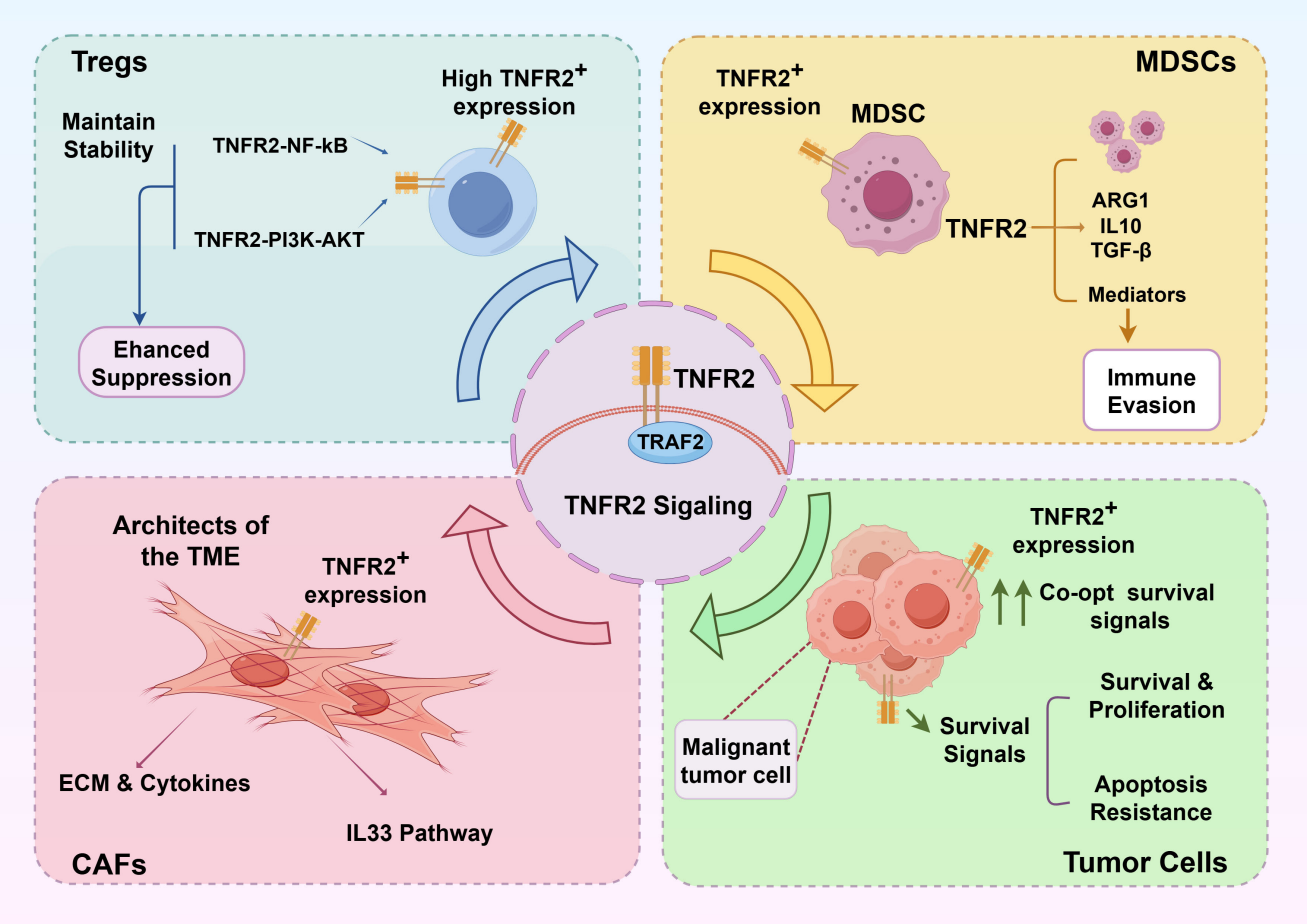
TNFR2-mediated multi-cellular crosstalk establishes a self-amplifying immunosuppressive network within the TME. **(A)** In Tregs, TNFR2 signaling stabilizes their immunosuppressive phenotype and enhances their suppressive capacity via the NF-κB and PI3K-AKT pathways. **(B)** In MDSCs, TNFR2 activation drives the production of immunosuppressive mediators, thereby facilitating tumor immune evasion. **(C)** Acting as the structural and chemical architects of the TME, CAFs utilize TNFR2 signaling to activate distinct pathways: the secretion of extracellular matrix (ECM) components alongside immunosuppressive cytokines, and the induction of the IL-33 pathway. These parallel mechanisms synergistically drive gastric cancer progression by enhancing EMT and cellular migration. **(D)** Malignant tumor cells upregulate TNFR2 to co-opt NF-κB-driven survival signals, leading to aggressive proliferation and profound resistance to apoptosis.

Following the recognition of the context-dependent roles of TNF, TNFR2 has attracted immense attention as a key mediator of immunosuppression within the TME (21, 22). Its restricted expression pattern positions TNFR2 as a critical regulator of the immunosuppressive niche (**Figure 2**). Elevated expression of TNFR2 across multiple cell types in the TME is strongly associated with shortened overall survival (OS) and progression-free survival (PFS) in cancer patients (23, 24).

### 3.1 Tregs and MDSCs

TNFR2 governs Treg homeostasis, phenotypic stability, and suppressive capacity through the activation of NF-κB and PI3K–AKT pathways (25, 26). Concurrently, TNFR2 signaling in MDSCs promotes the upregulation of immunosuppressive mediators, including arginase-1 (ARG1), IL-10, and TGF-β, facilitating their recruitment and reinforcing tumor immune evasion (27).

### 3.2 Cancer-associated fibroblasts and tumor cells

Beyond traditional immune suppressors, recent studies highlight that TNFR2 is profoundly expressed on CAFs and various tumor cells (28, 29). CAFs play structural and regulatory roles within the TME. Upon TNFR2 activation, they are stimulated to secrete various extracellular matrix components and immunosuppressive cytokines. Specifically, signaling through the TNFR2–NF-κB–IRF1 axis induces CAFs to release IL-33, thereby driving epithelial-mesenchymal transition (EMT) and enhancing the migration and invasion of gastric cancer cells (23, 30). Furthermore, many malignant cells directly upregulate TNFR2 to co-opt its NF-κB-driven survival signals, promoting aggressive proliferation and resistance to apoptosis (29, 30). The intense crosstalk among TNFR2^+^ tumor cells, CAFs, Tregs, and MDSCs forms a robust, self-amplifying immunosuppressive network that may contribute to the attenuation of anti-tumor immune responses.

## 4 Next-generation TNFR2-targeting strategies: monoclonal antibodies, ADCs, and bispecifics

Unlike global TNF blockade, which risks systemic infection and abolishes TNFR1-mediated tumor cell death, targeting TNFR2 selectively dismantles the TME’s defensive shield (11, 31). While early clinical evaluations focused on monoclonal antibodies (**Table 1**), the field is rapidly advancing toward more sophisticated multi-modal formats designed to enhance efficacy and overcome specific limitations (32). To highlight the rapid evolution of these multimodal and cell-type specific approaches, we have summarized the most promising strategies currently in preclinical development (**Table 2**), which aim to overcome the limitations of conventional monoclonal therapies through enhanced spatial and cellular precision.

**Table 1:** Clinical-stage of anti-TNFR2 antibodies in oncology.

Drug name	Developer	Antibody isotype/format	Clinical stage	Specific tumor indications
BI-1808	BioInvent	Human IgG1 (Ligand-blocking, Fc-enabled for ADCC)	Phase I/II	Advanced solid tumors, Cutaneous T-cell lymphoma (CTCL)
BI-1910	BioInvent	Human IgG1 (Non-ligand blocking)	Phase I/II	Advanced solid tumors (e.g., HCC, NSCLC)
LBL-019	Leads Biolabs	Humanized Monoclonal Antibody	Phase I/II	Advanced solid tumors, Refractory malignancies
HFB200301	HiFiBiO	Fully Human IgG1 (Enhanced cross-linking)	Phase I	Advanced solid tumors (e.g., Melanoma, NSCLC)
SIM-235	Simcere	Humanized IgG1	Phase I	Advanced solid tumors
NBL-020	NovaRock/CSPC	Fully Human Monoclonal Antibody	Phase I	Advanced solid tumors (often explored combo with PD-1)
AN3025	Adlai Nortye	Humanized Monoclonal Antibody	Phase I	Advanced solid tumors
BITR2101	Boston Immune (BITT)	Monoclonal Antibody	Phase I	Relapsed/Refractory CTCL, Non-Hodgkin Lymphoma

**Table 2:** Emerging preclinical modalities and cell-type specific TNFR2-targeting strategies.

Therapeutic modality	Representative format/targets	Key mechanism for cell-type specificity	Ref.
Bispecific Antibodies(Adaptive Checkpoint)	TNFR2 × PD-L1	TME Co-localization: Anchors TNFR2 blockade strictly at PD-L1-rich tumor sites to rescue exhausted CD8+ T cells without systemic toxicity.	(40)
Bispecific Antibodies(Innate Checkpoint)	TNFR2 × CD47	Avidity-driven Dual Blockade: Selectively binds dual-positive cells to dismantle TAM phagocytosis barriers and reverse adaptive immunosuppression.	(39)
Antibody-Drug Conjugates(ADCs)	Dual-targeting BsADCs (e.g., TNFR2 ×CCR8)	Payload Precision: Requires dual-antigen engagement to internalize and deliver cytotoxins strictly to highly immunosuppressive TME subsets.	(38)
Localized Delivery Vehicles	Armed Oncolytic Viruses (e.g., engineered AdV)	Spatial Confinement: Drives localized TNFR2 modulation and microenvironment remodeling exclusively within the viral-infected TME.	(39)

### 4.1 Monoclonal antibodies

Monoclonal anti-TNFR2 antibodies operate through dual mechanisms: directly antagonizing tmTNF-TNFR2 signaling to reverse Treg/MDSC suppressive functions, and utilizing Fc-dependent effector functions (such as ADCC and ADCP) to physically deplete TNFR2-high immunosuppressive cells (26, 33). The mechanistic foundation for these therapeutic approaches stems from seminal studies that unraveled the highly pleiotropic nature of TNFR2 within the TME. Early work by Torrey et al. established that antagonistic TNFR2 antibodies could simultaneously inhibit highly immunosuppressive Tregs and directly induce cell death in TNFR2-expressing tumor cells, primarily by blocking ligand binding and depriving them of essential NF-κB-driven survival signals (34). Adding a critical layer to this paradigm, Tam et al. demonstrated that dominant agonist antibodies targeting TNFR2 can provide potent Fc-dependent costimulation; this process relies on FcγR-mediated cross-linking to directly activate CD8^+^ T cells and promote robust, durable antitumor immunity (35). These dichotomous mechanisms—simultaneous Treg suppression and Fc-driven CD8^+^ T cell activation—collectively underscore why precise antibody engineering is absolutely essential to selectively harness TNFR2 biology without broadly disrupting immune homeostasis.

### 4.2 Antibody-drug conjugates and localized delivery vehicles

To leverage the dense overexpression of TNFR2 on Tregs and specific tumor cells, TNFR2-targeted ADCs are emerging as a promising strategy. By attaching a potent cytotoxic payload to an anti-TNFR2 antibody, ADCs can selectively deliver lethal doses of chemotherapy directly into the immunosuppressive TME. However, the applicability of ADCs faces distinct constraints: the payload must be carefully selected to avoid profound systemic immune depletion (on-target, off-tumor toxicity), as baseline TNFR2 expression is required for normal immune homeostasis and tissue repair (36). To mitigate these on-target, off-tumor toxicities, next-generation ADC designs are increasingly incorporating dual-targeting strategies. By engineering bispecific ADCs that pair a TNFR2-directed component with a specific “targeting arm” against a well-defined tumor-associated antigen (TAA), the cytotoxic payload can be selectively routed to malignant cells. This strict requirement for dual-antigen engagement ensures that cell death is restricted to the TME, sparing peripheral TNFR2+ immune populations. To circumvent these systemic constraints, recent strategies have utilized engineered oncolytic adenoviruses to confine TNF/TNFR2 blockade strictly within the TME, yielding potent local anti-tumor efficacy while mitigating the risks of systemic receptor suppression (37).

### 4.3 Bispecific antibodies

Bispecific antibodies represent another frontier, physically bridging TNFR2 blockade with other immunomodulatory pathways. Formats co-targeting TNFR2 and adaptive immune checkpoints (e.g., PD-1/PD-L1, CTLA-4), innate checkpoints (e.g., CD47), or Treg-associated targets (e.g., CCR8) are designed to simultaneously release immune brakes, profoundly deplete immunosuppressive populations, and aggressively drive localized T-cell activation (38–40). A critical engineering advantage of these bispecific formats is their capacity to achieve cell-type specificity through avidity-driven targeting. By strategically tuning the binding kinetics—pairing a high-affinity targeting arm against a TME-enriched checkpoint (e.g., CD47 heavily expressed on tumor-associated macrophages and malignant cells) with a functionally attenuated, lower-affinity TNFR2 arm—the therapeutic molecule selectively anchors only to cells co-expressing both targets. This mechanism prevents the systemic engagement of solitary TNFR2 on circulating leukocytes. Consequently, profound TNFR2 modulation, such as the depletion of Tregs or the targeted costimulation of terminally exhausted CD8+ T cells, is anatomically confined to the tumor site. For instance, novel PD-L1/TNFR2 bispecific antibodies have been shown to significantly increase tumor-specific targeting over monotherapies, comprehensively remodeling the TME by depleting Tregs and monocytic MDSCs (M-MDSCs) while rescuing CD8+ T cell exhaustion (40). Similarly, dual blockade formats targeting both TNFR2 and CD47 have demonstrated remarkable synergistic efficacy in dismantling both innate phagocytosis barriers and adaptive immunosuppression (39). The primary constraints hindering their rapid utilization include the complex engineering required to balance the binding affinities of the two arms, preventing steric hindrance, and managing the risk of hyper-inflammatory cytokine release syndrome (CRS) (41).

## 5 Challenges and future perspectives

While preclinical and early clinical data for TNFR2-targeted therapies are highly encouraging, successful translation hinges on managing the receptor’s pleiotropic biology. The fundamental hurdle remains achieving true tumor-specificity. Given that TNFR2 is essential for maintaining peripheral immune homeostasis, systemically depleting TNFR2-expressing cells with potent ADCs or enhanced-ADCC antibodies carries a severe risk of autoimmune-like toxicities. Navigating this narrow therapeutic window requires a profound shift in how these agents are engineered and delivered.

Moving forward, clinical success will rely heavily on robust patient stratification—specifically pinpointing those with immunologically “cold” tumors driven by dense networks of TNFR2^+^ CAFs and Tregs. Furthermore, rather than relying on simple receptor blockade, the field must aggressively exploit the distinct signaling kinetics of tmTNF versus sTNF. Ultimately, next-generation platforms like conditionally active biologics and rationally designed bispecifics are uniquely positioned to provide the spatial precision required to safely dismantle immunosuppression and conquer ICB resistance.
